# Comparative analysis of *Hmx* expression and the distribution of neuronal somata in the trigeminal ganglion in lamprey and shark: insights into the homology of the trigeminal nerve branches and the evolutionary origin of the vertebrate jaw

**DOI:** 10.1186/s40851-023-00222-9

**Published:** 2023-12-05

**Authors:** Motoki Tamura, Ryota Ishikawa, Yuki Nakanishi, Juan Pascual-Anaya, Makiko Fukui, Takashi Saitou, Fumiaki Sugahara, Filippo M. Rijli, Shigeru Kuratani, Daichi G. Suzuki, Yasunori Murakami

**Affiliations:** 1 Graduate School of Life and Environmental Sciences, University of Tsukuba, 1-1-1 Tennodai, Tsukuba, 305-8572 Ibaraki Japan; 2 Graduate School of Science and Engineering, Ehime University, 2-5 Bunkyo-cho, Matsuyama, 790-8577 Japan; 3 Department of Animal Biology, Faculty of Science, University of Málaga, Campus de Teatinos s/n, Málaga, 29071 Spain; 4 Department of Molecular Medicine for Pathogenesis, Ehime University Graduate School of Medicine, Toon, 791-0295 Japan; 5 Division of Biology, Hyogo Medical University, Nishinomiya, 663-8501 Hyogo Japan; 6 Evolutionary Morphology Laboratory, RIKEN Cluster for Pioneering Research (CPR), 2-2-3 Minatojima-minami, Chuo-ku, Kobe, 650-0047 Japan; 7 Friedrich Miescher Institute for Biomedical Research, Maulbeerstrasse 66, Basel, 4058 Switzerland; 8 University of Basel, Basel, Switzerland

**Keywords:** Lamprey, Shark, Trigeminal nerve, Craniofacial development, Evolution

## Abstract

The evolutionary origin of the jaw remains one of the most enigmatic events in vertebrate evolution. The trigeminal nerve is a key component for understanding jaw evolution, as it plays a crucial role as a sensorimotor interface for the effective manipulation of the jaw. This nerve is also found in the lamprey, an extant jawless vertebrate. The trigeminal nerve has three major branches in both the lamprey and jawed vertebrates. Although each of these branches was classically thought to be homologous between these two taxa, this homology is now in doubt. In the present study, we compared expression patterns of *Hmx*, a candidate genetic marker of the mandibular nerve (rV_3_, the third branch of the trigeminal nerve in jawed vertebrates), and the distribution of neuronal somata of trigeminal nerve branches in the trigeminal ganglion in lamprey and shark. We first confirmed the conserved expression pattern of *Hmx1* in the shark rV_3_ neuronal somata, which are distributed in the caudal part of the trigeminal ganglion. By contrast, lamprey *Hmx* genes showed peculiar expression patterns, with expression in the ventrocaudal part of the trigeminal ganglion similar to *Hmx1* expression in jawed vertebrates, which labeled the neuronal somata of the second branch. Based on these results, we propose two alternative hypotheses regarding the homology of the trigeminal nerve branches, providing new insights into the evolutionary origin of the vertebrate jaw.

**Supplementary Information:**

The online version contains supplementary material available at 10.1186/s40851-023-00222-9.

## Introduction

The jaw represents a major innovation in vertebrate evolution. With this predatory apparatus, jawed vertebrates achieved ecological success, resulting in their enormous diversification [1]. Despite considerable interest in elucidating the evolutionary origins of the vertebrate jaw, much about its emergence remains unclear [2–4].

The trigeminal nerve, the fifth cranial nerve (V), is an essential constituent of the jaw. It contains both motor and sensory components, enabling the apparatus to function as a whole. This peripheral nerve is also found in jawless vertebrates, including the sole extant lineage, the cyclostomes and has undergone substantial reorganization in association with the acquisition of the jaw.

As its name suggests, the trigeminal nerve is primarilycomposed of three main branches (rami) in jawed vertebrates: the ophthalmic, maxillary, and mandibular nerves (rV_1_, rV_2_, and rV_3_, respectively). Classically, the same nomenclature has been applied to the cyclostome lamprey [5], although this taxon lacks both maxilla (upper jaw) and mandible (lower jaw). However, recent studies have cast doubt on this homology, particularly for rV_2_ [6], as the fibers of the upper lip, which were traditionally recognized as “rV_2_” fibers, in larval lamprey contain motor as well as sensory components, while the gnathostome rV_2_ generally consists only of sensory fibers [6–8].

The sensory components of the trigeminal nerve, which play a role role equally important to that of the motor components, are often overlooked. The neuronal somata of the trigeminal sensory neurons are aggregated as an extramedullary ganglion (i.e., the trigeminal ganglion), so the distribution patterns of the neuronal somata in this structure may provide insights to infer the homology of the trigeminal nerve branches.

In the present study, we examined the distribution of the branch-specific neuronal somata in lamprey and shark, by studying the expression patterns of NK-like homeobox transcription factor *Hmx* genes, as *Hmx1* is expressed specifically in mouse rV_3_ neurons [9, 10]. We found peculiar patterns of both soma distribution and *Hmx* expression in the lamprey, while those in sharks were consistent with previous studies in mice. Based on these results, we propose two alternative hypotheses regarding the homology of the trigeminal nerve branches, and thus provide new insights into the evolutionary origin of the vertebrate jaw.

## Materials and methods

### Animals

#### Lamprey embryo collection

Adult male and female lampreys (*Lethenteron camtschaticum*) were collected in Miomote River, Niigata, Japan, during the breeding season (late May to June) from 2019 to 2021. They were brought into the laboratory and kept in aquaria with an enriched environment, and water was aerated and filtered continuously. Sexually mature lampreys were deeply anesthetized in 0.1% tricaine methanesulfonate (MS-222, Sigma) for artificial fertilization; mature eggs were squeezed from females and fertilized in vitro by sperm, and then kept in 1 × Steinberg solution at 8–12 °C. The resultant embryos were staged morphologically based on the method of Kuratani et al*.* (1997) [11]. Embryos were fixed with 4% paraformaldehyde in 0.1 M phosphate-buffered saline (PFA/PBS) at stages (st.) 26, 27 and 28. After fixation with 4% PFA/PBS at room temperature (RT) for 12 h, embryos were dehydrated in a graded methanol series (30%, 50%, 70%, 90% and 100%), and stored at − 25 °C.

### *Shark embryo* *collection*

Catshark embryos (*Scyliorhinus torazame*) were provided by RIKEN CDB. They were brought into the laboratory and kept in artificial seawater at 15 °C. They were staged morphologically based on the work of Ballard et al. (1993) [12]. Embryos were fixed with 4% PFA/PBS at st. 26, 27 and 28. After fixation with 4% PFA/PBS at room temperature (RT) for 12 h, embryos were dehydrated in a graded methanol series (30%, 50%, 70%, 90% and 100%), and stored at − 25 °C; and some embryos were washed with 1 × PBS, and stored at 4 °C without dehydrate.

### Isolation of cDNA clones of lamprey

We performed TBLASTN (v2.2.31 + ; [13]) searches of a previously published transcriptome assembly of *L. camtschaticum* [14] using the amino acid sequence of the following query proteins: *Hmx1* (GenBank: AF009367.1), *Hmx2* (GenBank: NP_666110.1) and *Hmx3* (GenBank: NM_008257.3) proteins of *Mus musculus*; *Hmx1* (GenBank: XP_002940264.2; Ensembl: ENSLACP00000013243), *Hmx2* (GenBank: XP_002937849.1; Ensembl: ENSLACP00000000977), *Hmx3* (GenBank: NP_001072829.1; Ensembl: ENSLACP00000001663) and *Hmx4* (*SOHo*) (GenBank: XP_002940265.1; Ensembl: ENSLACP00000012755) of *Xenopus tropicalis* and *Latimeria chalumnae*; *Hmx1* (GenBank: NP_001106998.1), *Hmx2* (GenBank: NP_001108570.2), *Hmx3a* (GenBank: NP_571709.2), *Hmx4* (*SOHo*) (GenBank: NP_001038836.2) of *Danio rerio*; and *Hmx* (JGI: 290,379) of the cephalochordate *Branchiostoma floridae*. The resulting transcripts were used as queries for searches in global protein databases at NCBI; only three resulting *Hmx* hits were retained: *HmxA*, *HmxB,* and *HmxC*. Protein alignment of the three resulting proteins showed a perfect match between homeobox sequence of all three paralogs. To perform specific in situ hybridization experiments, we subcloned regions excluding the homeobox by polymerase chain rection (PCR) using specific primers (*HmxA*, F: 5'- AAAGCGAGTAACCGAGCCAT-3' and R: 5'-GCTGTCGCCAATGGATTCTTC-3'; *HmxB*, F: 5'-CTCTGTTCCGTCGCCACATA-3' and R: 5'-TGGTGTCGATTGTTGTGGCT-3'; *HmxC*, F: 5'-GGCAATGACGGACAAGCAGTC-3' and R: 5'-TCTTCTTGGGGGGCGCA-3') and cDNA obtained from different lamprey stages (mixed Tahara Stages 18, 20, 22, 24, 26, 28). PCR fragments were checked by agarose gel electrophoresis for specificity, and the rest of the PCR reaction cleaned with QIAquick Gel Extraction Kit (QIAGEN) and cloned into pCRII-TOPO vector (ThermoFisher Scientific). Isolated clones were sequenced in-house at RIKEN BDR using an Applied Biosystems 3130xl DNA analyzer. Sequences of the three genes are provided in Supplementary File 1.

### Isolation of cDNA clones of catshark

*Hmx1* catshark homologs were isolated by PCR using st. 24 *S. torazame* cDNA as a template. Primers for PCR were designed on the *Hmx1* sequence of *S. canicula* (XM_038792135), which have been cloned previously [15]. These primer sequences were F: 5'-GGACGATGTGTTGGTGCTTATGG-3' and R: 5'-CAGAGCTGGCGAAGCTAACC-3'.

PCR product in the agarose gel was purified using QIAquick Gel Extraction Kit (QIAGEN) and DNA fragments were cloned using pGEM-T easy (pGEM-T Easy Vector Systems, Promega, Madison, USA). Isolated clones were sequenced using an Applied Biosystems 3730xl DNA analyzer at Macrogen, Japan. The isolated clone sequences were compared with the sequence of the orthologous gene of *S. canicula* registered in the NCBI database (XM_038792135.1). The sequence was registered in the DNA Data Bank of Japan (accession number: LC770109).

### Phylogenetic tree analysis of catshark *Hmx1*

Amino acid sequences were aligned using the FFT-NS-2 strategy from MAFFT v.7 and trimmed by trimAl. A phylogenetic tree was produced by the maximum likelihood method using RAxML version 8.2.12 [16], assuming the JTT model. A total of 1,000 bootstrap replicates was used to assess node confidence. Reference gene data are shown in Supplementary Table 1.

### Retrograde labeling of trigeminal neurons

#### Lampreys

Tetramethylrhodamine- and biotin-dextran conjugates (Invitrogen; D7162) were injected into the upper lip of live lamprey prolarvae to label the trigeminal nerve following the method described by Glover (1995) [17]. Injected prolarvae were incubated at RT for one hour to allow the dextran to label neurons retrogradely. Prolarvae were then washed with 10% Steinberg solution, and fixed in 4% PFA in PBS. The fixed specimens were dehydrated, clarified with LUCID [18], and then examined using a confocal laser microscope (Nikon A1R).

#### Sharks

Shark embryos were incubated under the conditions described above. At st. 30, they were fixed with 4% PFA/PBS at RT for 12 h. After fixation, the embryos were rinsed with 1 × PBS and stored at 4 °C. Then, small pieces of NeuroVue Red (Polysciences, Inc; 24835) and Jade (Polysciences, Inc; 24837) were inserted into the maxillary process and the mandibular process of the embryos, respectively. These specimens were incubated in 2% PFA/PBS at 37 °C for a month to allow retrograde labeling of neurons by NeuroVue. After incubation, the NeuroVue pieces were removed. The specimens were rinsed with 1 × PBS, clarified with LUCID, and then examined using a confocal laser microscope (Nikon A1R).

### Whole-mount in situ hybridization

Whole-mount in situ hybridization was performed following the method described by Murakami et al. (2001) [19].

Antisense RNA probes were transcribed using T7 or SP6 RNA polymerase (Roche) in conjunction with digoxigenin conjugated dUTPs (Roche, 11277073910) following standard protocols. Specimens were treated with a mixture of hydrogen peroxide and methanol (1:5) overnight for bleaching, and were rehydrated in PBS containing 0.1% Tween 20 (PBT). Samples were digested with 10 mg/ml proteinase K (Invitrogen, AM2546), post-fixed for 20 min with 4% PFA/PBT containing 0.2% glutaraldehyde, and then washed with PBT and prehybridized in hybridization buffer (50% formamide, 5 × SSC, 1% SDS, 1% Blocking Reagent (Roche, 11096176001), 50 µg/ml heparin sulfate, 5 mM EDTA, 0.1% CHAPS) for 90 min at 70 °C. The specimens were then incubated in a hybridization buffer with 0.1 mg/ml DIG-labeled RNA probe (Roche, 11277073910) for 48 h at 70 °C. After hybridization, the specimens were washed twice in 50% formamide, 5 × SSC, and 1% SDS for 30 min at 70 °C, and the solution was substituted gradually with 10 mM Tris–HCl (pH 7.5) containing 0.5 M NaCl and 0.1% Tween 20 (TBST). RNaseA was added to a final concentration of 0.05 mg/ml and the specimens were incubated for 30 min at RT. The samples were washed twice with 2 × SSC in 50% formamide for 30 min at 70 °C, twice in 2 × SSC containing 0.3% CHAPS for 30 min at 70 °C, and twice in 0.2 × SSC containing 0.3% CHAPS for 30 min at 70 °C. For immunological detection, the embryos were blocked with TBST containing 0.5% blocking reagent (Roche, 11277073910) for 90 min, and incubated with alkaline phosphatase (AP)-conjugated anti-digoxigenin Fab fragments (diluted 1:4000; Roche 11093274910), at 4 °C overnight. The specimens were washed ten times for 30 min each in TBST at RT. Alkaline phosphatase activity was detected with NBT/BCIP in NTMT (100 mM Tris HCl pH 9.8, 100 mM NaCl). Stained specimens were fixed in 4% PFA/PBS.

### Section in situ hybridization

Shark embryos stored in 100% methanol at − 25 °C were rehydrated in a graded methanol series (90%, 70%, 50%, 30% methanol in PBT) and PBT. The specimens were replaced in a graded series of sucrose (12.5%, 25%). Then, samples were embedded in Tissue-Tec O.C.T. Compound (Sakura Finetek, Japan), and stored at − 80 °C. Frozen Sects. (20 µm) were prepared using a cryostat (Leica CM30505S).

Antisense RNA probes were transcribed using T7 or SP6 RNA polymerase (Roche) in conjunction with digoxigenin conjugated dUTPs (Roche, 11277073910) following standard protocols. Specimens were washed in PBT. The samples were digested with 10 mg/ml proteinase K (Invitrogen, AM2546). They were post-fixed for 20 min with 4% PFA/PBT, then washed with PBT, and prehybridized in HYB mix (10% salt solution (2 M NaCl, 0.1 M Tris HCl pH 7.5, 0.05 M NaH_2_PO·H_2_O, 0.05 M Na_2_HPO_4_, 0.05 M EDTA), 50% formamide, 5% dextran sulphate, 1% Denhardt solution) for two hours at RT. The specimens were then incubated in a hybridization buffer with 0.1 mg/ml DIG-labeled RNA probe (Roche, 11277073910) for 12 h at 65 °C. After hybridization, the specimens were washed four times in Wash Solution (50% formamide, 1 × SSC, 0.1% Tween20) for 30 min at 65 °C. For immunological detection, embryos were blocked with TBST containing 0.5% blocking reagent (Roche, 11277073910) for 90 min, and incubated with AP-conjugated anti-digoxigenin Fab fragments (diluted 1:4000; Roche 11093274910) at 4 °C overnight. The specimens were washed ten times for 30 min each in TBST at RT. Alkaline phosphatase activity was detected with NBT/BCIP in NTMT (100 mM Tris HCl pH 9.8, 100 mM NaCl). Stained specimens were fixed in 4% PFA/PBS.

### Whole-mount immunostaining

Immunostaining with anti-acetylated tubulin monoclonal antibody (Sigma, T6793) or HuC/HuD antibody (Invitrogen, A-21271) was performed according to the method described by Kuratani et al. (1997) [11] with some minor modifications as described below. The samples were soaked in a 10:1 mixture of 30% H_2_O_2_ water and 100% methanol and put under a fluorescent light for 12 h at RT for bleaching. After 12 h, embryos were washed in TBST containing 5% dimethyl sulfoxide (TSTd) for three hours at RT. After washing, the samples were sequentially blocked with 5% nonfat dry milk in TSTd (TSTM). This was followed by incubation in the primary antibody (1:1000 in TSTM) and DAPI (D9564, 1 mg/mL; Sigma-Aldrich) for three days at RT. After washing with TSTd, samples were incubated with secondary antibody (life technologies, Alexa fluor 488, A-21422) diluted 1:500 in TSTM for two days. After a final wash in TSTd, the embryos were dehydrated, clarified with a 1:2 mixture of benzyl alcohol and benzyl benzoate (BABB), and then examined under a microscope (Zeiss AXIO Imager. AI, Zeiss Lumar. V12).

### In situ hybridization combined with immunostaining

Whole-mount in situ hybridization was performed as described above. Subsequently, the samples were washed several times with TSTd. Neuronal somata were visualized by immunostaining as described above.

## Results

### Morphological observations of the trigeminal nerve

**Fig. 1:**
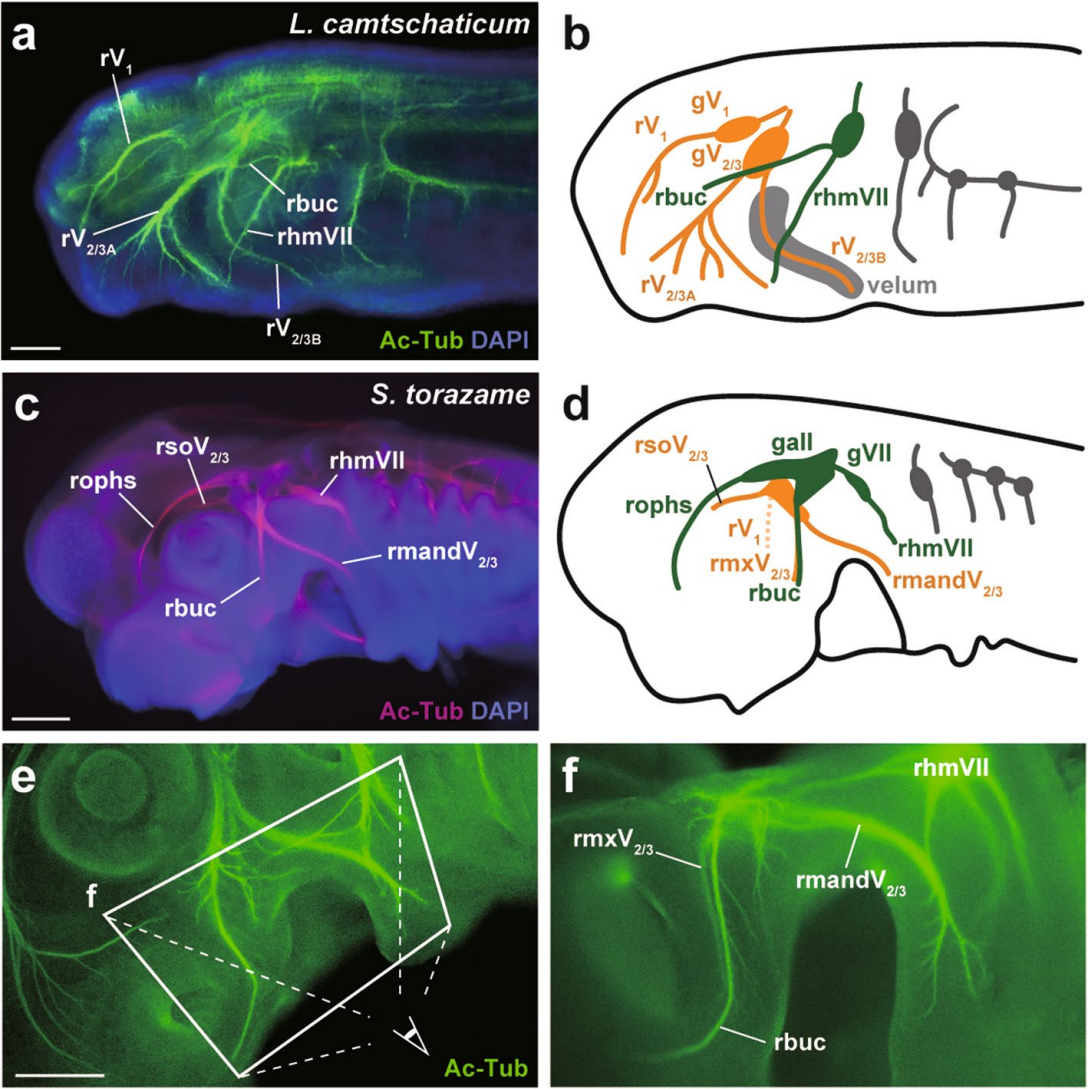
Morphology of the trigeminal nerve. **a** Whole-mount immunofluorescence for the Arctic lamprey (*L. camtschaticum*) prolarva at st. 27. Peripheral nerves are visualized by an anti-acetylated tubulin antibody in green and cell nuclei are labeled DAPI in blue. **b** Schematic illustration of the cranial nerves. **c** Whole-mount immunofluorescence for the catshark (*S. torazame*) embryo at st. 27. Peripheral nerves are visualized by an anti-acetylated tubulin antibody in magenta, and cell nuclei are labeled by DAPI in blue. **d** Schematic illustration of the cranial nerves. **e**, **f** Another catshark embryo (st. 30) for detailed microscopy. Peripheral nerves are visualized by an anti-acetylated tubulin antibody in green. The overview of the ventral head regions is shown in (e). The magnified oral region is shown in (f), observed from a slightly ventral aspect. Scale bars: 100 μm for (a), 1 mm for (c), and 1 mm for (d)

To compare the morphologies of the trigeminal nerve between lampreys and sharks, we first performed immunofluorescence analysis with anti-acetylated tubulin antibody to visualize peripheral nerves (Fig. 1).

In the Arctic lamprey *L. camtschaticum*, we confirmed that the trigeminal nerve has three main branches, as has been reported in gnathostomes generally. Although the homology of the anteriormost branch or ramus (i.e., the ophthalmic nerve, rV_1_) is widely accepted, the homology of the other two branches is in doubt [11, 20, 21]. Therefore, we used the term “rV_2/3A_” for the second branch and “rV_2/3B_” for the third branch in the lamprey, according to Oisi et al. (2013) [20]; rV_2/3A_ mainly innervated the upper lip region, whereas rV_2/3B_ innervated the velar region (Fig. 1a, b).

The trigeminal ganglion of the lamprey was composed of two main parts. The anterior part was the ophthalmic ganglion (gV_1_), which consisted of the somata of rV_1_. The posterior part contained the somata of both rV_2/3A_ and rV_2/3B_. Reluctantly, we retain the traditional name, the “maxillomandibular” ganglion (gV_2/3_), despite its misleading implications.

In gnathostomes, the remaining two branches of the trigeminal nerve other than rV_1_ are generally called the maxillary and mandibular nerve (rV_2_ and rV_3_, respectively). In the catshark *S. torazame*, however, an additional prominent branch (i.e., the supraoptic branch, rsoV_2/3_), distributes its fibers superficially over the dorsal eye region [22]. Here, we have followed the abbreviations for the trigeminal nerve branches of the catshark proposed by Kuratani et al*.* (2000) [22], using rmxV_2/3_ and rmandV_2/3_ instead of rV_2_ and rV_3_, respectively.

In catshark embryos at st. 27 and st. 30, the trigeminal and facial/lateral line nerves were distributed adjacent to each other (Fig. 1c–f). In particular, the buccal nerve (rbuc), a branch of the anterior lateral line nerve, was overlying rV_2_ (the same branch was similarly observed in the lamprey, as shown in Fig. 1a, b). In addition, the ganglion of the facial nerve (i.e., the geniculate ganglion, gVII) was fused with the anterior lateral line nerve ganglion (gall).

The following experiments were conducted based on these observations and nomenclature.

### Identification and expression analysis of lamprey *Hmx* genes

To elucidate the homology of the trigeminal branches between cyclostomes and gnathostomes, we focused on *Hmx* genes encoding NK-like homeobox transcription factors, because mouse *Hmx1* is expressed specifically in the neuronal somata of rV_3_ [10].

We found three *Hmx* homologs in the genome of the Arctic lamprey *L. camtschaticum*, consistent with a previous report regarding the sea lamprey *Petromyzon marinus* [23]. Based on their sequence similarity, we named the three genes *HmxA*, *HmxB*, and *HmxC*. We next performed whole-mount in situ hybridization analysis to examine the expression patterns of these genes.

**Fig. 2:**
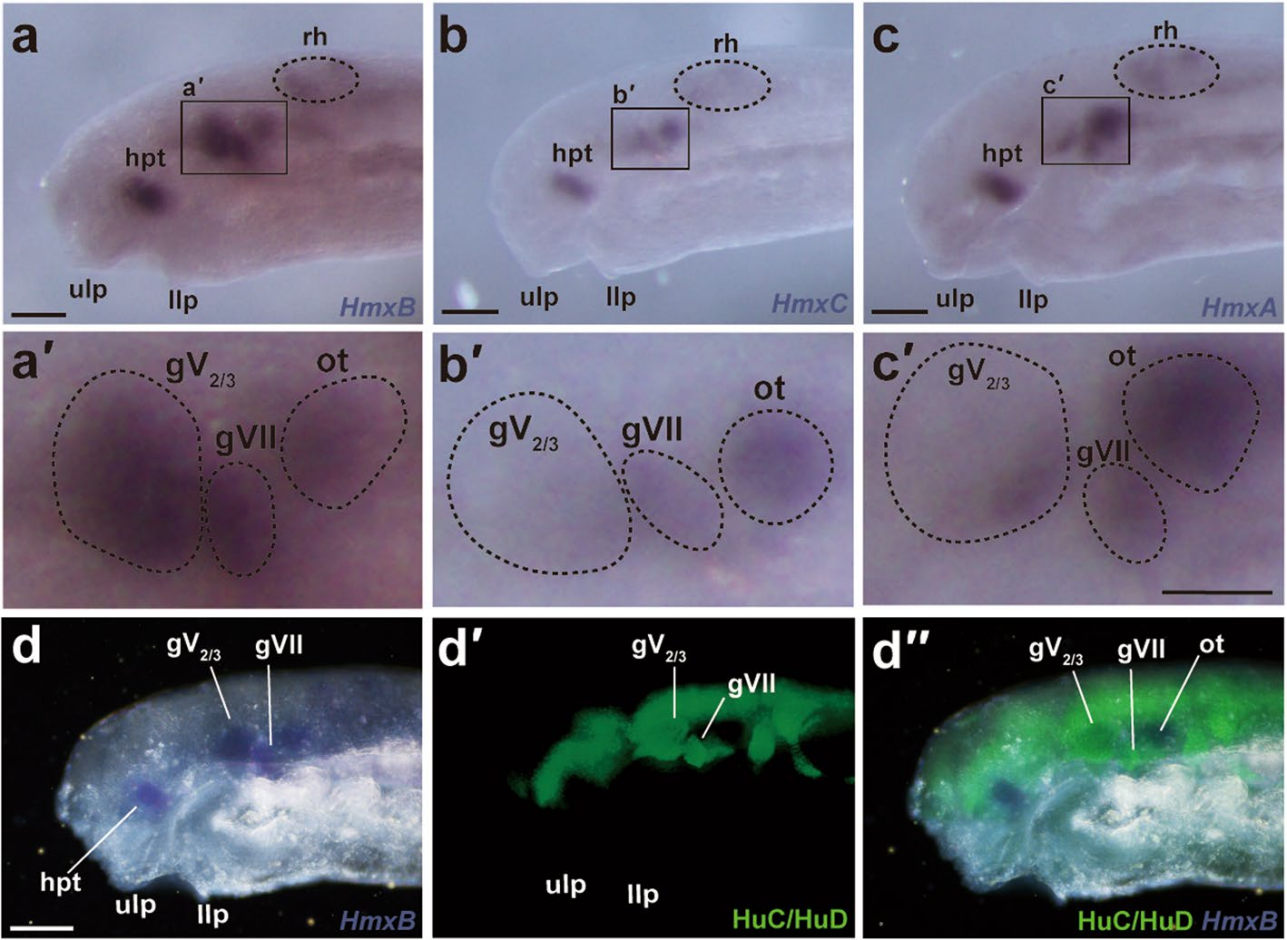
Expression patterns of the lamprey *Hmx* genes. **a**, a′ Expression of *HmxB* at st. 27 in the head region (a) and its magnified preotic region (a'). **b**, b′ Expression of *HmxC* at st. 27 in the head region (**b**) and the magnified preotic region (b'). **c**, c′ Expression of *HmxA* at st. 27 in the head region (c) and the magnified preotic region (c'). **d**–d′′ Double staining of *HmxB* in situ hybridization and HuC/HuD immunofluorescence; *HmxB* only, HuC/HuD only, and merged images are shown in (d), (d′), and (d′′), respectively. Scale bars: 100 μm for (a), (b), (c) and (d) and 50 µm for (a′), (b′) and (c′)

**Fig. 3:**
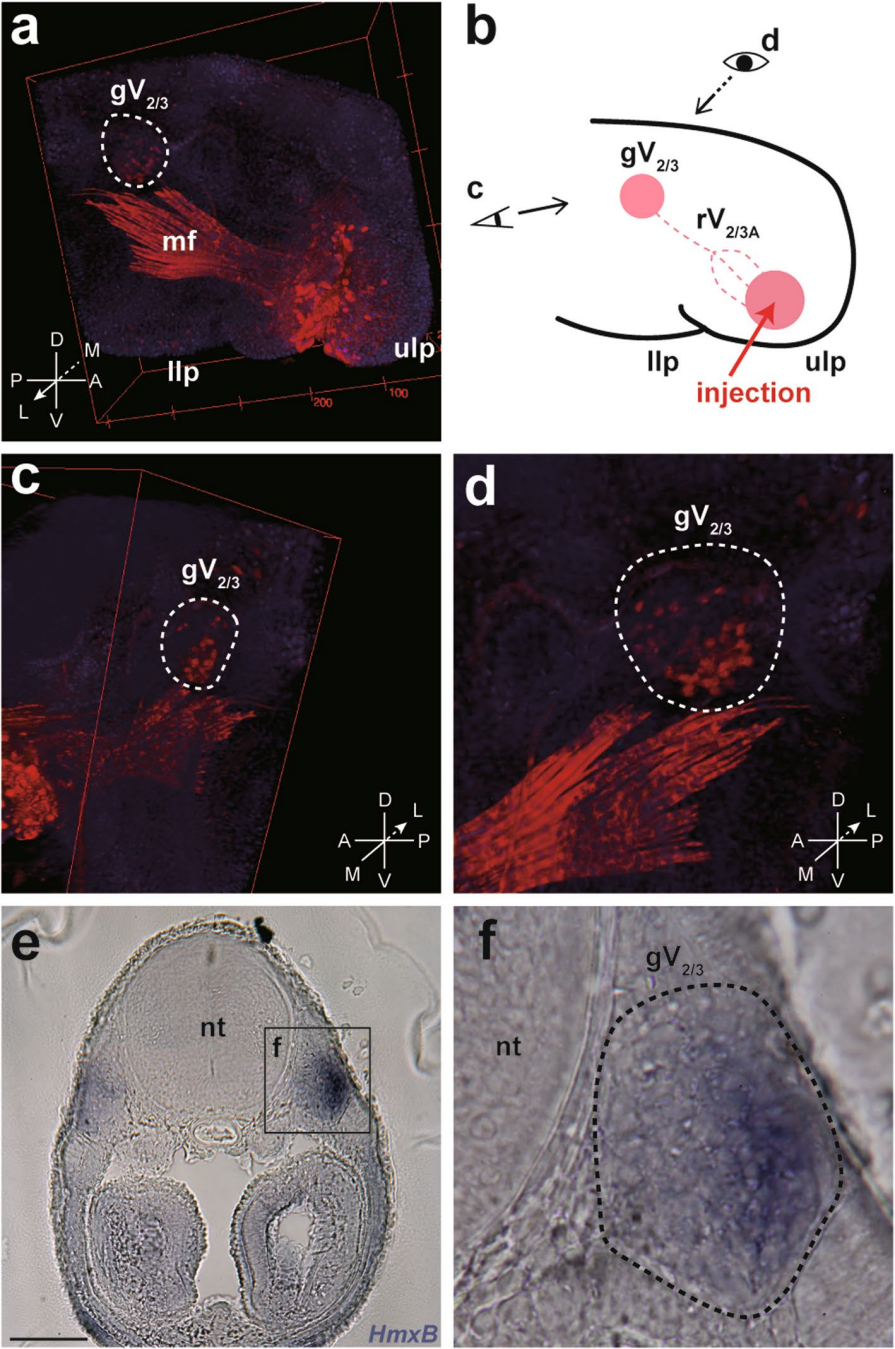
Distribution of the rV_2/3A_ neuronal somata in the gV_2/3_ in the lamprey. **a**, **b** Confocal microscopy of a labeled prolarva at st. 30 (**a**) and its schematic illustration (b). Rhodamine-conjugated dextran is injected into the upper lip (ulp). As a result, some neuronal somata in the gV_2/3_ are retrogradely labeled. Some muscle fibers (mf) are unexpectedly labeled as well. **c**, **d** Reconstructed 3D image viewed from caudal (**c**) and left sides of the specimen (**d**). Note that labeled somata are distributed predominantly in the lateral and ventrocaudal parts of the gV_2/3_. **e**, **f** Whole-mount in situ hybridization of *HmxB* in a transverse section at the level of the gV_2/3_ at st. 28 (**e**) and the magnified gV_2/3_ region (**f**). Scale bars: 50 µm for (e)

In the head region of lamprey prolarva at st. 27, *HmxB* was expressed in the hypothalamus, gV_2/3_, gVII, otic capsule, and some regions of the rhombencephalon (Fig. 2a). In particular, the gene was expressed uniformly throughout the entire gV_2/3_ as seen in lateral views of whole-mount specimens (Fig. 2a′). Nevertheless, analysis of frozen sections showed that the expression was localized only on the distal side of the ganglion (Fig. 3e, f). We also confirmed that *HmxB* was expressed in some sensory ganglia (i.e., gV_2/3_ and gVII) by observing colocalization of the immunofluorescence signals of the HuC/HuD antibody (Fig. 2d), which labels developing neuronal somata [21].

Similarly, *HmxC* was expressed in the hypothalamus, gV_2/3_, gVII, otic capsule, and some regions of the rhombencephalon at st. 27 (Fig. 2b). Its relative expression level in gV_2/3_ compared to other regions (e.g., the hypothalamus or gVII) was weaker than that of *HmxB* (Fig. 2a′, b′). Moreover, *HmxC* appeared to be expressed more strongly in the ventral than the dorsal part of gV_2/3_, forming an expression gradient (Fig. 2b′).

*HmxA* was expressed in the hypothalamus, gV_2/3_, gVII, otic capsule, and some regions of the rhombencephalon at st. 27 (Fig. 2c). Thus, its overall expression pattern was also similar to *HmxB* and *HmxC*. However, the *HmxA* expression in gV_2/3_ was restricted to the ventrocaudal part, distinct from the other two *Hmx* genes (Fig. 2c′).

In summary, we found that all lamprey *Hmx* genes were expressed in gV_2/3_. However, their expression patterns in gV_2/3_ were quite different: *HmxB* was expressed entirely on the lateral side, *HmxC* showed a dorsoventral gradient, and *HmxA* expression was restricted only to the ventrocaudal part of gV_2/3_. These results suggest that each of the *Hmx* gene marks specific neuronal populations.

### Distribution of rV_2/3A_ neuronal somata in the gV_2/3_ in lamprey prolarvae

To compare the expression patterns of lamprey *Hmx* genes with the distribution of the neuronal somata of the trigeminal nerve branches in gV_2/3_, we performed retrograde labeling of rV_2/3A_ nerve fibers. As the upper lip of the lamprey is exclusively innervated by rV_2/3A_ [11], we injected rhodamine-conjugated dextran into this region at st. 30. We then examined the labeled neurons by confocal microscopy to analyze their spatial distribution.

The results showed that the neuronal somata of rV_2/3A_ were predominantly distributed in the lateral and ventrocaudal parts of the gV_2/3_ (Fig. 3a–d). As described above, *HmxB* showed the broadest expression pattern among the three lamprey *Hmx* genes (Fig. 2). Nevertheless, its expression was restricted in the lateral and ventrocaudal part of gV_2/3_ (Fig. 2a′ and Fig. 3e, f), where the labeled neuronal somata of rV_2/3A_ were found. This comparison between the distribution of the labeled neurons and the expression pattern of the lamprey *Hmx* genes thus suggests that the neuronal somata of rV_2/3A_ express at least *HmxB*.

These experiments showed that *Hmx* genes were expressed in some parts of the lamprey gV_2/3_ in a manner similar to mouse *Hmx1* expression. These findings suggest that *Hmx* genes mark a specific population of trigeminal neurons, and can therefore be used for comparison between cyclostomes and gnathostomes. For this purpose, however, it should first be confirmed that the rV_3_-specific (for sharks, rmandV_2/3_-specific) expression pattern of *Hmx1* is conserved in the gnathostomes.

### Phylogenetic analysis of shark *Hmx* genes

As *Hmx1* expression specific to the rV_3_ neuronal somata has been reported only in mice, it is unclear whether similar it shows similar expression patterns in other gnathostomes. Therefore, we examined sharks, which belong to the cartilaginous fishes (i.e., chondrichthyans), the basal-most extant lineage of the gnathostomes.

**Fig. 4:**
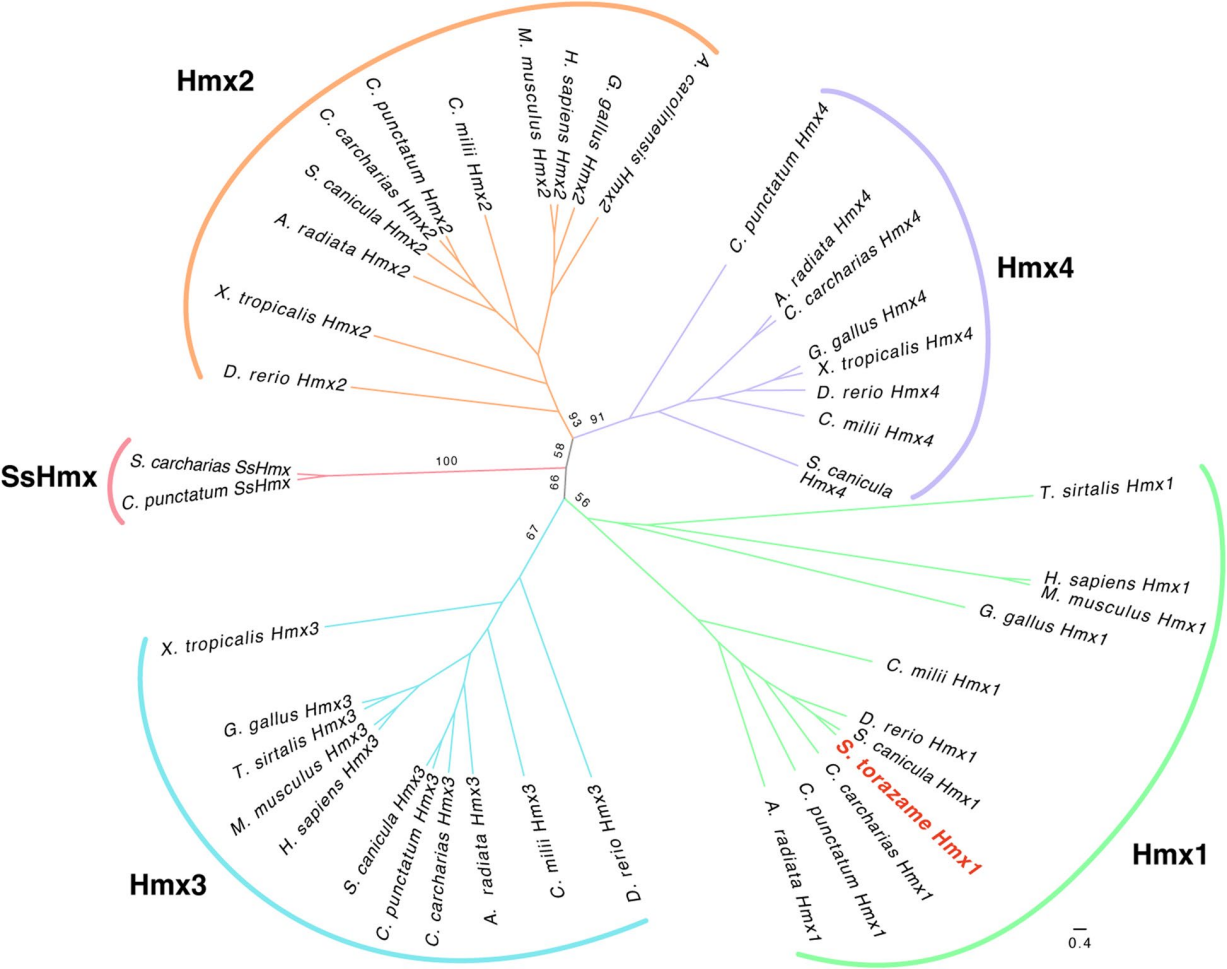
Molecular phylogenetic tree for shark *Hmx* genes. The tree was constructed using the Maximum Likelihood method. The numbers at the nodes represent bootstrap values. *A. carolinensis*, *Anolis carolinensis*; *A. radiata*, *Amblyraja radiata*; *C. carcharias*, *Carcharodon carcharias*; *C. milii*, *Callorhinchus milii*; *C. punctatum*, C*hiloscyllium punctatum*; *D. rerio*, *Danio rerio*; *G. gallus*, *Gallus gallus*; *H. sapiens*, *Homo sapiens*; *M. musculus*, *Mus musculus*; *S. canicula*, *Scyliorhinus canicula*; *S. torazame*, *Scyliorhinus torazame*; *T. sirtalis*, *Thamnophis sirtalis*; *X. tropicalis*, *Xenopus tropicalis*

From the transcriptome data of the small-spotted catshark *Scyliorhinus canicula*, one of the closest relatives of *Scyliorhinus torazame*, we found five candidate *Hmx* genes. Based on phylogenetic analysis, four of these genes were classified as *Hmx1*, *Hmx2*, *Hmx3*, and *Hmx4* (Fig. 4). Interestingly, the remaining gene formed a cluster with undefined *Hmx* genes found only in sharks (*Chiloscyllium punctatum*, *Rhincodon typus*, *S*. *canicula*, and *S*. *torazame*), not in rays or holocephalans; we named these shark-specific *Hmx* (*SsHmx*) genes.

Based on these results, we examined the expression pattern of shark *Hmx1* to examine the conservation of *Hmx1* expression in gV_2/3_.

### Expression analysis of the shark *Hmx1* gene

We isolated a candidate *Hmx1* gene from the transcripts of *S*. *torazame* embryos. As phylogenetic analysis revealed that it showed close affinity to the known *Hmx1* genes (Fig. 4), it was named *Hmx1*. To examine the expression pattern of *Hmx1*, we performed whole-mount in situ hybridization analysis.

**Fig. 5:**
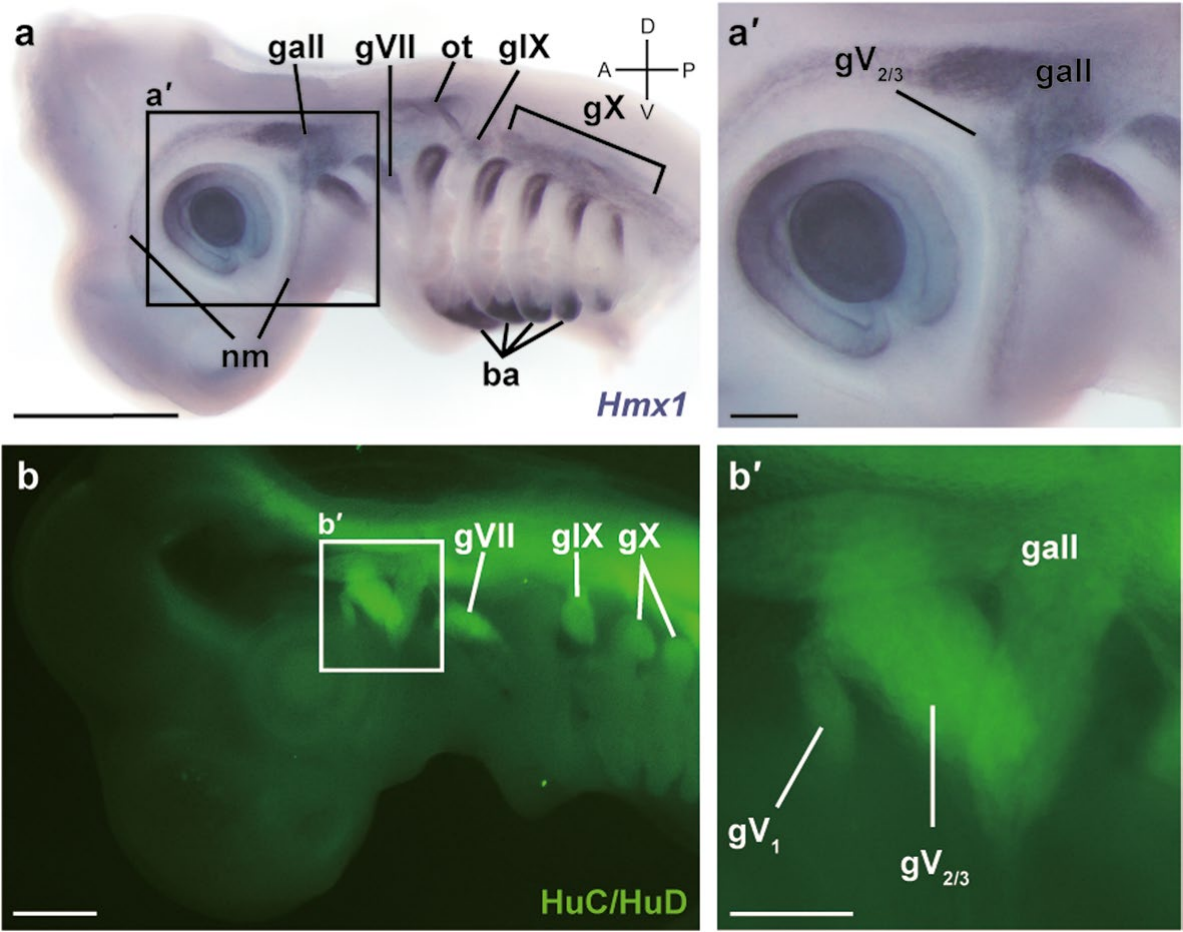
Expression pattern of the shark *Hmx1* gene. **a**, a' Expression of *Hmx1* at st. 27 in the head region (a) and the magnified preotic region (a'). **b**, b' Immunofluorescence for the HuC/HuD antibody at st. 27. Cranial sensory ganglia are visualized. Scale bars: 1 mm for (a), 200 µm for (a'), 500 µm for (b) and 200 µm for (b')

**Fig. 6:**
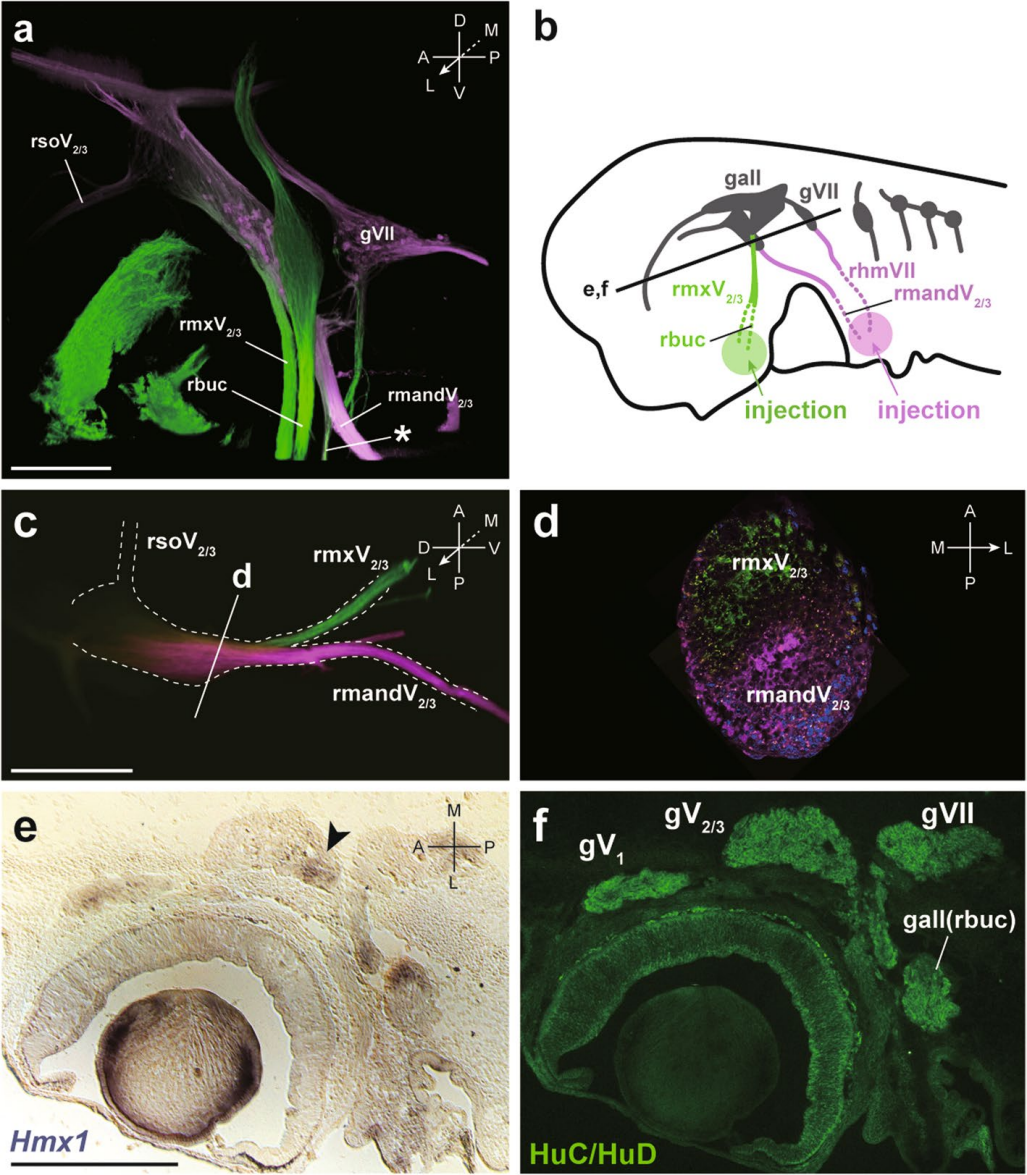
Distribution of the branch-specific neuronal somata in the gV_2/3_ in the shark. **a**, **b** Confocal microscopy of a labeled embryo at st. 30. (a) and its schematic illustration (b). NeuroVue Jade (green) and Red (magenta) are inserted into the maxillary process and mandibular process, respectively. As a result, NeuroVue Jade labeled the rmxV_2/3_, rbuc, a part of the rmandV_2/3_, and some portion of rhmVII, while NeuroVue Red marked the main branches of the rmandV_2/3_ and rhmVII. The asterisk (*) indicates the maxillary nerve of the rmandV_2/3_. **c**, **d** 3D reconstruction (c) and histological analysis (d) of another specimen, indicating that the neuronal somata of rmxV_2/3_ and rmandV_2/3_ are distributed in the rostral and caudal parts of the gV_2/3_, respectively. **e**, **f** Double staining of the shark *Hmx1* section in situ hybridization and HuC/HuD immunofluorescence in a horizontal section at the level of gV_2/3_. The arrowhead indicates that *Hmx1* is expressed in the caudal part of the gV_2/3_

At st. 27, shark *Hmx1* was expressed in the retina, anterior lateral line ganglion, neuromasts, gV_2/3_, gVII, otic capsule, and both dorsoventrally distal parts of the gill arches (Fig. 5a, a′). However, whole-mount immunofluorescence analysis using anti-HuC/HuD antibody showed that the anterior lateral line ganglion overhung gV_2/3_ (Fig. 5b, b′), suggesting that it would be difficult to conduct detailed analysis of the expressing region inside gV_2/3_ by whole-mount in situ hybridization. To resolve this issue, we performed in situ hybridization of *Hmx1* combined with HuC/HuD immunohistochemistry on sections at st. 30, and confirmed that *Hmx1* was expressed in the posterior part of gV_2/3_ (Fig. 6e, f).

These results suggest that shark *Hmx1* is expressed in branch-specific neuronal somata, possibly that of rmandV_2/3_, in gV_2/3_. However, the branch-specific distribution in gV_2/3_ remained unknown in sharks. Therefore, it was unclear whether *Hmx1* was expressed in the neuronal somata of rmandV_2/3_, which prompted us to perform neurolabeling experiments for sharks as described below.

### Distribution of branch-specific neuronal somata in the gV_2/3_ in shark embryos

To clarify the branch-specific distribution pattern of the sensory neuronal somata, we retrogradely labeled trigeminal nerve branches by inserting two pieces of differently colored NeuroVue tracing filters (NeuroVue Jade and Red) into the maxillary process and mandibular process, respectively.

Using confocal microscopy, we found that NeuroVue Jade labeled the rmxV_2/3_, rbuc, a part of the rmandV_2/3_, and a portion of rhmVII, while NeuroVue Red marked the main branches of rmandV_2/3_ and rhmVII (Fig. 6a, b). Furthermore, 3D reconstruction and histological analysis revealed that the neuronal somata of rmxV_2/3_ and rmandV_2/3_ were distributed in the rostral and caudal parts of gV_2/3_, respectively (Fig. 6c, d).

Taking into account the expression pattern of shark *Hmx1* (Fig. 6e, f), these results support the hypothesis that *Hmx1* is expressed specifically in the rV_3_ neuronal somata in sharks. In conclusion, our findings suggest that *Hmx1* expression specific to the rV_3_ neuronal somata is conserved throughout the gnathostomes, while lampreys show different expression patterns.

## Discussion

### Significance of the trigeminal nerve in the evolution of the vertebrate jaw

The vertebrate jaw is embryologically derived from the first pharyngeal arch, which is also present in cyclostomes. Given that the earliest vertebrates had no jaw, the evolution of the jaw must have involved changes in developmental mechanisms that underlie preexisting structures in jawless vertebrates [4]. Such changes would have included rewiring of the peripheral nerve (i.e., the trigeminal nerve) for precise sensing and responses of the jaw region. That is, the transformation of the trigeminal nerve provides important insights to understand the evolution of the jaw, one of the most innovative apparatuses acquired in the lineage of the gnathostomes.

To clarify the evolutionary modification of the trigeminal nerve, it is necessary to assess the homology of its branches between jawless vertebrates (or cyclostomes as their extant representatives) and jawed vertebrates. For this purpose, “nerve–muscle specificity” [24] may be useful, although the validity of this concept has been questioned several times [25, 26]. Specifically, the transcription factor *Engrailed* is expressed in the levator arcus palatini (a mandibular arch muscle) of actinopterygians [27] and in the primordium of the lamprey velum [28], suggesting that these two structures are homologous [29]. It is also known that the levator arcus palatini and the velum are innervated by the motor components of the gnathostome rV_3_ and lamprey rV_2/3B_, respectively [7, 29]. If we accept nerve–muscle specificity as a valid concept, we can thus deduce that these two branches are homologous.

However, this reasoning ignores the fact that these peripheral nerves are composed of mixtures of motor and sensory fibers. Here, the nerve–muscle specificity cannot be applied to sensory nerves, as they extend not only to muscles (or more precisely, muscle spindles) but also many other organs and tissues; a different strategy is thus required to examine the homology of sensory components of the trigeminal nerve. In the present study, we focused on the expression of *Hmx* genes in the trigeminal ganglion, as *Hmx1* is a marker of the neuronal somata of rV_3_ in mice [9, 10]. By comparing their expression patterns and the distribution of the sensory neuronal somata in the trigeminal ganglion, we can now reevaluate the homology of the trigeminal nerve branches, providing a new perspective on the evolutionary origin of the vertebrate jaw.

### Conserved expression of *Hmx1* in the trigeminal ganglion in sharks

To make a phylogenetic comparison of *Hmx* expression between the lamprey and gnathostomes, it is first necessary to confirm that *Hmx1* expression in the neuronal somata of rV_3_ is actually conserved among the gnathostomes. For this purpose, we focused on the chondrichthyans (sharks and rays), the basal-most extant group of jawed vertebrates.

There are four *Hmx* subfamilies in the gnathostomes, *Hmx1*, *Hmx2*, *Hmx3*, and *Hmx4* (also called sensory organ homeobox protein gene, *SOHo*). In this study, we found all four paralogs, as well as a shark-specific *Hmx* (*SsHmx*), from the transcriptome data of *S*. *canicula* (Fig. 4). We further found that *S*. *torazame Hmx1* was expressed in the caudal part of gV_2/3_, where the neuronal somata of rmandV_2/3_ are distributed (Figs. 5 and 6). These results strongly suggest that *Hmx1* is also expressed in the neuronal somata of rmandV_2/3_ (i.e., rV_3_) in chondrichthyans.

It is notable that the distribution patterns of the neuronal somata in the trigeminal ganglion show variation in different lineages. For example, the neuronal somata of rV_1_, rV_2_, and rV_3_ are aligned along the rostrocaudal axis in the mouse trigeminal ganglion [9], while the neuronal somata of rV_3_ are located rostral to those of rV_2_ with some overlap in chicks [30]. Nonetheless, this dissimilarity appears to be due to lineage-specific modification in birds, as the soft-shelled turtle shows the same pattern as seen in mice [30]. Teleosts and sharks also show the mouse-type organization, suggesting that this is an ancestral condition ([31]; this study). Another inconsistency found in the gnathostomes is that *Hmx1* is not expressed in the trigeminal ganglion in zebrafish and medaka [32], but this appears to be an actinopterygian/teleost-specific feature because its expression has been reported in other jawed vertebrate lineages (mouse, [33]; chick, [34, 35]; *Xenopus*, [36]; shark, this study).

In summary, there are some differences in the trigeminal ganglion organization and *Hmx1* expression in the gnathostomes, but the present findings provide a reasonable basis to support the likelihood that *Hmx1* expression in the neuronal somata of rV_3_ is generally conserved in this group.

### Peculiarity of *Hmx* gene expression and soma distribution in the trigeminal ganglion in the lamprey

By contrast, the lamprey showed some idiosyncrasies with regard to both *Hmx* gene expression and soma distribution in the trigeminal ganglion.

As reported previously [23], lampreys have three *Hmx* paralogs (*HmxA*, *HmxB*, *HmxC*). The phylogenetic analysis by Papadogiannis et al. (2022) [23] indicates that lamprey *HmxB* is the closest to the gnathostome *Hmx1/3*, whereas lamprey *HmxA/C* forms a sister group with gnathostome *Hmx2/4*. In contrast, the relationship according to genomic position suggests that lamprey *HmxA/B* and *HmxC* are paralogous to gnathostome *Hmx1/3* and *Hmx2/4*, respectively [23, 32]. Therefore, although lamprey *HmxB* has the closest affinity to gnathostome *Hmx1/3*, the orthology of *Hmx* genes between the lamprey and gnathostomes remains unclear.

Interestingly, we found that all three lamprey paralogs are expressed at least in a part of gV_2/3_ (Fig. 2). In the gnathostomes, *Hmx4* is expressed in the *Xenopus* trigeminal ganglion in addition to *Hmx1*, although *Hmx4* has been lost in mammals [23, 36, 37]. Previous studies have shown that *Hmx2* and *Hmx3* are not expressed in the trigeminal ganglion in gnathostomes [33], suggesting that all *Hmx* paralogs were expressed in the trigeminal ganglion in the common ancestor of the vertebrates and then *Hmx2* and *Hmx3* expression were secondarily lost in the gnathostome lineage. In fact, the upstream regulatory network of *Hmx* for cranial ganglion development can be traced back to protochordates, which lack any cranial ganglion, but which have one *Hmx* gene expressed in bipolar tail neurons (i.e., cells thought to be homologues of the neural crest cells) [23].

Although the expression patterns of the lamprey *Hmx* paralogs show substantial differences, they are commonly expressed in the lateral and/or ventrocaudal parts of the gV_2/3_ in a manner similar to the expression of mouse *Hmx1* (Figs. 2 and 3e, f). However, these parts are not occupied by the neuronal somata of rV_2/3B_, which is generally considered a homologous branch to the gnathostome rV_3_, but instead by those of rV_2/3A_ (Fig. 3). This suggests two alternative hypotheses regarding the homology of the trigeminal nerve branches.

### *Hmx* gene expression and homology of the trigeminal nerve branches

#### The “rV_2/3B_ = r﻿V_3_” hypothesis

**Fig. 7:**
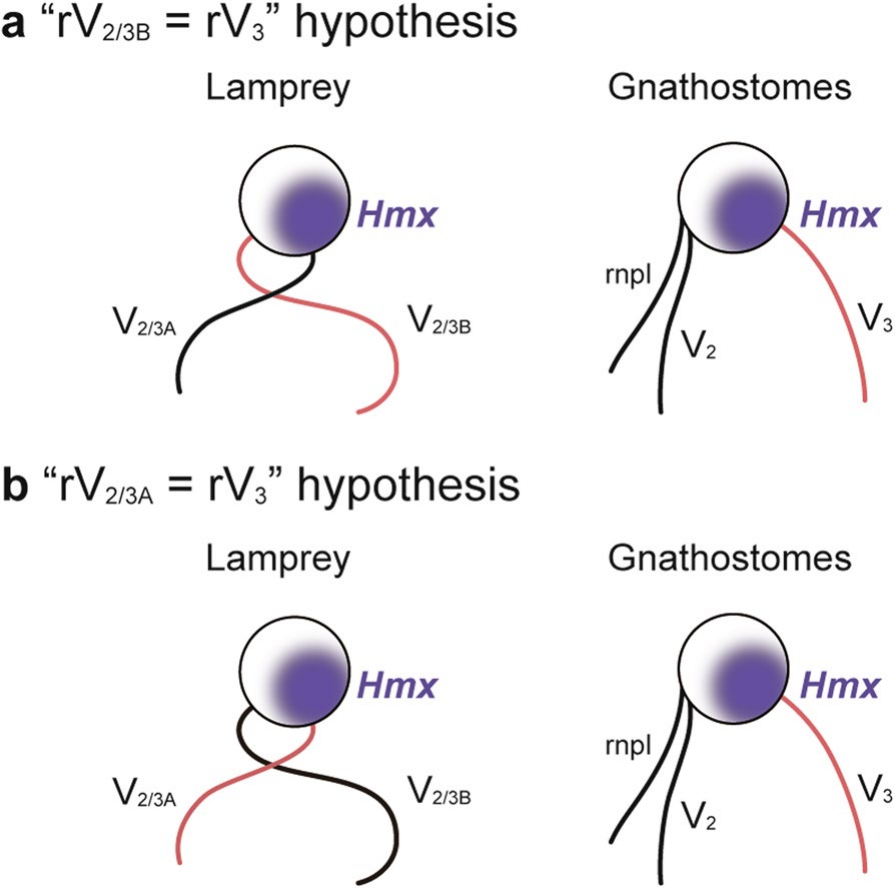
Schematic diagram for the evolution of the trigeminal nerve branches. **a** “rV_2/3B_ = rV_3_” hypothesis, where the lamprey rV_2/3B_ is regarded as homologous to the gnathostome rV_3_. **b** “rV_2/3A_ = rV_3_” hypothesis, where the lamprey rV_2/3A_ and the gnathostome rV_3_ are thought to be homologous. The gnathostome counterpart of the lamprey rV_2/3B_ may have been lost in the gnathostome lineage

One hypothesis to explain the inconsistency between the expression patterns of *Hmx* genes and the organization of the trigeminal ganglion is that *Hmx* genes do not mark the neuronal somata of rV_3_ and its counterpart in the lamprey, but are actually involved in subregional (e.g., rostrocaudal) patterning in the trigeminal ganglion. Therefore, the gnathostome rV_3_ and lamprey rV_2/3B_ can be taken to be homologous (Fig. 7A).

This hypothesis is consistent with the classical theory on the homology of rV_3_ based on motor nerve innervation [5] and the velar origin hypothesis of the vertebrate jaw [29] described above. In fact, a loss-of-function mouse mutant of *Hmx1* does not show any effect on the trigeminal ganglion and nerve [10]. Furthermore, as noted above, *Hmx1* is not expressed in the trigeminal ganglion in zebrafish and medaka [32]. These lines of evidence suggest that *Hmx1* may not be essential for the identity of and axon guidance for rV_3_ neurons in the gnathostomes. Similarly, lamprey *Hmx* genes may not specify rV_2/3B_ neurons but simply define the lateral and/or ventrocaudal parts of gV_2/3_.

However, if lamprey rV_2/3B_ is indeed homologous to gnathostome rV_3_, then the distribution of the homologous neuronal somata of this branch should have changed markedly during vertebrate evolution. Although the anteroposterior position of the rV_2_ and rV_3_ neuronal somata has been changed in the avian lineage [30], the change in distribution from lamprey to vertebrate does not appear plausible, as the organization of the trigeminal ganglion is generally conserved except for this bird-specific modification.

Furthermore, even if the gnathostome *Hmx1* has no role in the formation of the trigeminal ganglion and nerve, it is still possible that the rV_3_ neurons maintain the expression of this gene. In fact, Hodge et al*.* (2007) [9] showed that retrograde BMP signaling regulates trigeminal sensory neuron identities, and disruption of this signaling pathway results in expansion of *Hmx1* expression into the maxillary region and a part of the ophthalmic region of the trigeminal ganglion in mice. These findings indicate that *Hmx1* does mark rV_3_ neurons, at least in mice.

#### The “r﻿﻿V_2/3A_ = r﻿﻿V_3_” hypothesis

Based on the assumption that *Hmx1* can be used as a marker gene for rV_3_ neurons, we alternatively hypothesized that the expression of gnathostome *Hmx1* and its lamprey homologs designate homologous branches. This hypothesis suggests the possibility that the lamprey counterpart of the gnathostome rV_3_ is not rV_2/3B_, as commonly posited, but rV_2/3A_ (Fig. 7b).

Classically, the lamprey rV_2/3A_ and rV_2/3B_ had been considered homologous simply to the gnathostome rV_2_ and rV_3_, respectively (e.g., Johnston, 1905 [5]). In contrast to this traditional view, Higashiyama and Kuratani (2014) [6] showed that the gnathostome rV_2_ has two components, the nasopalatine and maxillary (or more precisely, palatoquadrate) nerves, with the former possibly corresponding to the lamprey rV_2/3A_ and the latter being a gnathostome novelty. This discovery also prompted us to question the postulated homology of the lamprey rV_2/3B_ and the gnathostome rV_3_, as it has been accepted without serious examination.

Interestingly, lamprey rV_2/3A_ contains motor components innervating the mandibular arch-derived upper lip muscles, while no motor fibers are found in the gnathostome rV_2_ with the possible exception of holocephalians [38–40]. The motor fibers in the gnathostome rV_3_, which innervates mandibular arch muscles, may thus correspond to at least a part of the motor component of the lamprey rV_2/3A_. This correspondence is further supported by the observation that the motor nuclei of the gnathostome mandibular nerve and the lamprey rV_2/3A_ are both located in rhombomeres 2 and 3 [41].

As the lamprey rV_2/3B_ passes through the velum, a pumping apparatus, it appears to be involved in the control of this organ. There is some debate whether the earliest jawless fishes also had the velum, or whether this organ is a synapomorphy of the cyclostomes [28]. As the gnathostomes lack any velum-like organ for ventilation, this structure may have been lost in the gnathostome lineage or newly acquired in the common ancestor of the cyclostomes. The same may hold for the trigeminal nerve branch corresponding to the lamprey rV_2/3B_, suggesting that there are no rV_2/3B_ counterparts in the gnathostomes.

Nevertheless, it remains unclear how the neuronal population in the *Hmx*-negative anterodorsal part of gV_2/3_ can be characterized. Kuratani et al. (2004) [39] labeled the neurons in this part by injecting tracer into the lamprey lower lip, arguing that these labeled cells were rV_2/3B_ neurons. Together with our hypothesis that rV_2/3A_ is homologous to rV_3_, this would mean that the *Hmx*-negative anterodorsal part of gV_2/3_ is not homologous between lamprey and gnathostomes. However, there is also a branch of rV_2/3A_ that innervates the lower lip [20, 29], which implies the possibility that Kuratani et al. (2004) [39] actually labeled this lower lip-innervating rV_2/3A_, not rV_2/3B_. To characterize the neuronal population and discuss its homology in this *Hmx*-negative part of gV_2/3_, further detailed research on the distribution of rV_2/3B_ and lower lip-innervating rV_2/3A_ neurons is required.

## Conclusions

We examined *Hmx* gene expression in the lamprey and shark trigeminal ganglion compared to the distribution of the sensory neuronal somata in this structure. Our results suggest that rV_3_-specific expression of *Hmx1* is generally conserved among the gnathostomes. In addition, we found that *Hmx* genes were also expressed in the lamprey trigeminal ganglion and rV_2/3A_ neuronal somata were distributed in the *Hmx*-positive region. Based on these results, we proposed two hypotheses regarding the homology of the trigeminal nerve branches, namely, that the gnathostome rV_3_ is homologous to the lamprey rV_2/3A_ and rV_2/3B_, respectively. Further studies, for example, comparative examination of the subregional neuronal organization and gene expression profiles in the trigeminal ganglion, are needed for detailed discussion.

### Supplementary Information

**Additional file 1.**

**Additional file 2.**

## Data Availability

Not applicable.

## Abbreviations

hpt: Hypothalamus
gall + gVII: Anterior lateral line and geniculate ganglion
gV_1_: Ophthalmic ganglion of the trigeminal nerve
gV_2/3_: Maxillomandibular ganglion of the trigeminal nerve
llp: Lower lip
mf: Muscle fibers
ot: Otic capsule
rh: Rhombencephalon
rbuc: Buccal nerve
rophs: Superficial ophthalmic branch of the anterior lateral line nerve
rV1: Ophthalmic nerve
rV_2_/rmxV_2/3_: Maxillary nerve
rV_3_/rmandV_2/3_: Mandibular nerve
rV_2/3A_: Upper-lip-projecting branch of the trigeminal nerve
rV_2/3B_: Velum-projecting branch of the trigeminal nerve
rhmVII: Hyomandibular branch of the facial nerve
ulp: Upper lip
