# Supplementary material for: Comparative analysis of Hmx expression and the distribution of neuronal somata in the trigeminal ganglion in lamprey and shark: insights into the homology of the trigeminal nerve branches and the evolutionary origin of the vertebrate jaw
Source: Zoological Lett. 2023 Dec 5;9:23. doi: 10.1186/s40851-023-00222-9 (PMC10696661; doi:10.1186/s40851-023-00222-9)
Supplement: Supplementary file 1 — Additional file 1. [file 40851_2023_222_MOESM1_ESM.docx]

**Supplementary File X**: Hmx sequences of the Arctic lamprey, *Lethenteron camtschaticum*, found in this study. Sequences highlighted in light blue represent unstranslated regions (UTR); Underlined sequences correspond to the clones used in this study.

>Lethenteron camtschaticum HmxA

CACAAATTGGCGCCGGGCTGACGCTCCGCTCCTGTCTGGGACGTGCAAGTTGGCGAGATTCGCTCACCCCGTTCGCTCGCTCGCTCCCACTCACCCGCTCGCTCGCTCTCTCGCTCGCTCCCACTCAAGAGCGAGCAAAGCGAGTAACCGAGCCATGTTCACCGCCCTCTCTCGTCTCGCGAGCCGCGCCACTGCTCCCTAGAACAGCTCCGCCTCGGAGGGAGAGCAAGGAGAGAGAGAGTAGAGAGAGAGAGAGAGCGGGGAGAGAGAAGAAAGAGGAGAGAGAGAGAGGAAAAAAACATCCAAGCGAGAGGGAAAGATCGACATCGCCGATGCGCCTTGGACACGCTTTTCTAAGCTCCGCGCTGGCATTTCTTATTTAGGCGCTATTCGTCGCGTGGAGTCGAATTCATTTTTTTTGTTGTCTTTACTCCGCACACAGAGAGACCGAGGCGTCGCCACGGGACGAAGAAAAAGATGTCCGAGAAAGCGACCACGCCGCAAAATCCCGGCCCCAACAAAGTGTCATCGTTCTTCATCCAAAATCTTCTCAATTCCGAAGACAAGCCCGCGACCAAGCCCGAGCGCCAGCTCATCTGTTTCGGATTCGGCAATGTGCGCTTCGGCGAGGAGATGTCGGCTCTCGGGCACGGCGCGGGCCTCGTGGTGGCTCCGTTCGAGATCCCCATGCCGAGATTCGCAATGCCGCCCTTTCGCCTCGTCGAGAAGTCCATTGCCCCGTGGCACCCCTACCTCCCGTTCGGACAGACGGAGAGTCCTAGGGGATGCTCCCCGTCTCTGCCCAACAGTGATCACGCCTCGCCGTCTCCCTTCAGCGACCGGGGCTCTCCCGGCACCGCGGCCAATAAATGCGAGGACGCGGACGGGGGAGACTCGTCGAGGGGCGCCGATGAGCAACTCACGCTGCACAAAACTGCCGCCGCCGCAGCCGCCGGTGCAACCGAAGACGCAGCAGACGCCAAGGAAGAATCCATTGGCGACAGCGGCTCCGAGCGACAACTCGAGACGAGCGGCGGCAGCTGCGGCGGCGGCGGCGGCGTCGATCTTCTGGAGAAGAAGGGCGGACGCAAGAAGAAGACGCGGACCGTGTTCTCTCGGAGCCAAGTGTTCCAGCTGGAGTCGACGTTCGACATGAAGCGCTACCTGAGCAGCGCGGAGCGCGCGGGTCTCGCCGCGTCGCTGCACCTCACCGAGACGCAGGTGAAGATCTGGTTCCAGAACCGGCGCAACAAGTGGAAGCGCCAGCTGGCCGCCGAGCTCGAGGCCGCCAACCTTTCGCACACGGCGCAGCGCCTGGTCCGGGTGCCCATCTTGTACCACGAGAGTTCAGGG

>Lethenteron camtschaticum HmxB

GGGGTGACCCGAACGTGCGTGTAATAATTACTTCTTGAGCAAAAAAAAGAGCGCCACACGGGGCGTATGCATACGCGATTGTTGTGATAGTAAGAGGCCGACGTAGCAAAAACACGCGGCCGCATCGTCCGTGTGTACATAAGAGAGAGAGAGAAAAAGGAGGAAGCGATACCACGCATGGAGCCCAGGGAAAACGCGGCCAAGAGGCCAGGTCACTCGCTCTCCAAATTCACCATCCGATGCATCTTGGGAGCCCAGACGGACGGGAGCGACTGCGGCAGCGAGGATGAGAGCGACGCGGACGGGAGGAGGAGGGGGACCCCTGCCGCCACTCAGGCTCCGGGAGGGGGCGCCGCCGCCGCCGCTGCCGCCGCCGCCGCCACAGTGGCTCATGATCGACATCGCACGGGCTCCTCCCGTCTCTCTGAGCCCTTGGAGGGGTCTCGCAAAGTCGCCTGCTGGCTGCAGGAACACCAGATCGCTTGCTGCCACGAAGTGGCCAAGCGAGCATTGGCCGGGCACGGCGTCTCTCAGGAGAGGATCCAACCCCAACTACAGCAGCAGCAGCAGCAGCCGCCGCCGCCGCATCAGCAGCATCAGCAGCAGCATCACCATCCGCATCAGGAGGCGAGGGCCGGTCACGTCACCTCCCCAGCGCACACGGAGCCGCGCAGCAGTAACAGCAGCAGCAGCATCGTCATCGACACTCCGCCGGCGCTGTCGGCAACCGCGCCTCGGTTAAACGGGACAACCGCGCCCCACGTTGTTGTTGTTGGTGGCGGCGACGAAGATGACGACGATGATGACGATGACGACGACGACGATGACGGCGGCGGCGGCGGTGGCGGTGGCATCGTCGTGAGCGGGGACAGGGCCTGCGAGAGCCCCCCTCAGCACGGCAGCCCGCGTGCGCCCCCCAAGAAGAAGACGCGGACCGTGTTCTCTCGGAGCCAAGTGTTCCAGCTGGAGTCGACGTTCGACATGAAGCGCTACCTGAGCAGCGCGGAGCGCGCGGGTCTCGCCGCGTCGCTGCACCTCACCGAGACGCAGGTGAAGATCTGGTTCCAGAACCGGCGCAACAAGTGGAAGCGCCAGCTGGCCGCCGAGCTCGAGGCCGCCAACTTGGCGCAGGTGTCGGCGGCGCACAGACTCGTGCGCGTGCCCGTGCTCTACCGGGAAGGGGGCTTGCACGGGAGAGCGGCTCCCGCGGGAGGAGTCAGAGCCCCGCTCGCGTTCCCCTACCCCTTCTACTACCCCGGCGGTGCATTGACACACTTCGCGATGCCCTACGCCGCCAGCAGCTCGGGCCTCATGCAGTGAGAGTGTGGACAAAACTCGTTGCTCACCACGCGTTGTTGTTGTTGTTGGGGGTGTGGGTGTAGAAGCAGAGGGCTCTGTTCCGTCGCCACATATACGAGTGTGGTAATGATACGTTTTAGTCCGTCAGCCTGTCGTTGGCTTTTCAGACCAATGTTATTAATCGTCCAGCAGCAGCAGAATTCGTTTTGAAGCGAAGCAAACGTCTGATGCGGTTTCTTCGACGTTTGTGCGTCTCGTCGGCAGTCGTGCCCCCGTGCTTGCGGATGAGTGCGCGCCCGGACAGCGAGACTGACAGACGACGAGACAGCGTCGAAAACTCACGCGAACCCCCATCGTCAGTTGTGTGCGAGGTGATACAGACGCGATCCATTTGTGGATACCCCACTGACTTGTAATGACGCCCGATTCTCAACAGGTGATCGAATGTTGTTGCGAAAGCGATGGCTCCGTATCTTGCAGAGTGTTAACAGATGTACTAAGTGTGTCGTTGAGTCTGATGGGAGACGCGTAGAGGCTTTGAAATGTAATGTTGTTGTTTTTTTATCAATGTAACGTGTCTCCATTGTTTTAGTGGGGTAGCTATAAATCGATCACCCTTGTATGATTTCCACGACTGTGTACTGCCGCTTTTTGTCCATCCGCTGCTGGATGACTGCCTATTCTTTGGCCACACTTCACCGCGCTGGTCAGTGCAGAGTTGATGAAAGCGGGGAGAAGCATACAAGTGAGGGCATAGTGCAGTACAACATGTGCGAAAATTATATATCTGCAAGTTTTAGTCTACATACTGTACTGCATATGTACATATACTGAATAATACTGTATTTGTGCATATACTGTATGTCATTGGTTCTCAACCGGGGTGGGGTGTGGGGGGGGGGGTGTGCGCAGGCTACGGCACGGATACCGTGCAGGACAAAACGCTGAATGTGCGCGGAGAAACGTGAACGTTGCGACGTCAAGCCACAACAATCGACACCATGAAACAAGTGTGAGGCTGCGGCG

>Lethenteron camtschaticum HmxC

GCAGGCGCGCCTTTATGCCCGCGGGGCAATGACGGACAAGCAGTCCCCGCCGTGTAACGCGGCGCCCAAGTTCAGCATCCAGCGCATCCTAGGCACGGACCTGGCCGACTCGTGCAGGAGACCGAGCCACGGCGCCATCAAGGGCGACGCGTGGAGAGGCTTCCCGCTGTCCGCGGCCGGCGTCGACCTCTCCCTGTTCTCGACGAGCAACGGGCCCCACTACGCGACGGGCGCCAGGGAGGCGCTCTGCGGCTGTAAGAAGAAGCGGCTCCAGGCAGCAGCGACAACTCCCCCCGCCGCCGGACGGTGCCGCCCCGCGCAGGCAGCGGCGGACATCGATGCGCTCGTGTCGCCCGCGTCAAGCGTCTCGTCTGACGCCACCAAAGAGACGTCCTTAGATTTATCAAACCCGGACGACGTCGCCGAAAGGATCTCGCCGGACTTCAAAGCCCCGCCGCAGAAGCGCTCGGTGGGTCGGGTCACGGCGGACGACATAGACTCCCAGGCGGAGGAGAGAGACGCGGAGAGCGCCGCGTGTCGGGCCGGGGACAGGGCCTGCGAGAGCCCCCCTCAGCACGGCAGCCCGCGTGCGCCCCCCAAGAAGAAGACGCGGACCGTGTTCTCTCGGAGCCAAGTGTTCCAGCTGGAGTCGACGTTCGACATGAAGCGCTACCTGAGCAGCGCGGAGCGCGCGGGTCTCGCCGCGTCGCTGCACCTCACCGAGACGCAGGTGAAGATCTGGTTCCAGAACCGGCGCAACAAGTGGAAGCGCCAGCTGGCCGCCGAGCTCGAGGCCGCCAACTTGGCGCAGGTGTCGGCGGCGCACAGACTCGTGCGCGTGCCCGTGCTCTACCGGGACGCGAGCCTCCTGCGCGCGGCGGCTGCAGCCTCGCTGCCCCTGCCCGGTGCCCTCTGCTTCCCGGGGGCCGGACTCTCCCAGTTCCCCACTTCGTTCCCCAGCAACCTCATCTGA
